# Vestibular Functioning in Children with Neurodevelopmental Disorders Using the Functional Head Impulse Test

**DOI:** 10.3390/brainsci10110887

**Published:** 2020-11-20

**Authors:** Simona Caldani, Moetez Baghdadi, Ana Moscoso, Eric Acquaviva, Christophe-Loïc Gerard, Vincenzo Marcelli, Hugo Peyre, Paola Atzori, Richard Delorme, Maria Pia Bucci

**Affiliations:** 1 MoDyCo, UMR7114, CNRS Paris University, 92001 Nanterre, France; simona.caldani@gmail.com (S.C.); moetez.baghdadi@gmail.com (M.B.); 2 EFEE Centre for Functional Exploration of Balance in Children, Robert Debré Hospital, 75019 Paris, France; 3 Child and Adolescent Psychiatry Department, Robert Debré Hospital, 75019 Paris, France; ana.moscoso@gmail.com (A.M.); eric.acquaviva@aphp.fr (E.A.); cl.gerard@gmail.com (C.-L.G.); hugo.peyre@gmail.com (H.P.); paola.atzori@gmail.com (P.A.); richard.delorme@aphp.fr (R.D.); 4 Department of Otolaryngology, Audiology and Vestibular Unit, ASL-NA1, Ospedale del Mare, 80147 Naples, Italy; vincenzo.marcelli@gmail.com; 5 Départment D’études Cognitives, Université de Paris, 75005 Paris, France; 6 Human Genetics & Cognitive Function, Institut Pasteur, 75015 Paris, France

**Keywords:** vestibular and visual systems, atypical brain development, neurodevelopmental disorders, etiology

## Abstract

Several studies in children with neurodevelopmental disorders (NDDs) including autism spectrum disorders (ASDs), reading impairment, or attention deficit/hyperactive disorder (ADHD) pointed toward a potential dysfunction of the vestibular system, specifically in its complex relationship with the cerebellum. The aim of the present study was to test the functional vestibulo-ocular reflex (VOR) responses in children with NDDs to measure functional performance of the vestibular system. The VOR is specifically involved in this stabilization of the image on the retina during rapid movements of the head. To perform this study, four groups of children with ASD, ADHD, reading impairment, and with neurotypical development (TD) were enrolled (*n* = 80). We performed the functional head impulse test (fHIT), which measured the percentage of correct responses by asking the child to identify an optotype briefly presented during passive head impulse in each direction of each semicircular canal plane. We observed significantly lower correct answers in children with NDDs compared with those with TD (*p* < 0.0001). Surprisingly, there was no significant difference between the three groups of children with NDDs. Our study fostered preliminary evidence suggesting altered efficiency of vestibular system in children with NDDs. VOR abnormalities estimated using the fHIT could be used as a proxy of NDD impairments in children, and represent a potential biomarker.

**Highlights** The vestibulo-ocular reflex (VOR), which is specifically involved in the stabilization of the image on the retina during rapid movements of the head, can be explored using the functional head impulse test (fHIT). The number of appropriate responses during the fHIT was significantly lower in children with neurodevelopmental disorders (NDDs) than in children with neurotypical development. Surprisingly, we did not report any significant difference when exploring the VOR functioning between all the three subgroups of children with NDDs. The VOR abnormalities we observed in children with NDDs brought further evidence of the strong relationship between a loop involving the vestibular system, the ocular system and the cerebellum, and atypical brain development.

## 1. Introduction

Vision plays a key role in the knowledge of our environment during each moment of our daily life. It is essential for the coordination of all our movements in natural space, for the precision to perform a task, to use a tool, as well as for reading or writing, and is fundamental in relationships and social communication with other humans. Eighty percent of the information coming from the world goes through our eyes and is immediately processed by the brain. The stabilization of an image on the retina mainly depends on the activity of the vestibular and visual systems [1]. The activity of each of these two systems depends on the oscillation frequency of the head. For instance, at low frequencies (<0.1 Hz), the visual system is predominant; at medium frequencies, the vestibular and visual systems interact together to stabilize the gaze; while at high frequencies (from 1 to 5 Hz), only the vestibular system enters into action [1].

When the head rotates rapidly in the horizontal or in the anterior or posterior planes, the two semi-circular canals of each pair participate in the estimation of the speed of the head movement. The ipsilateral canal at rotation gives the main excitatory information—via the vestibular nerve fibers—to which the weaker and inhibitory information from the contra-lateral channel is added. In response, the oculomotor system will induce an eye movement equal in amplitude, but in an opposite direction to the movement of the head. This reflex, called the vestibulo-ocular reflex (VOR), allows the stabilization of the image on the retina during rapid movements of the head and is measurable during a test known as the head impulse test (HIT) [2].

The VOR is monitored at the central level [1] and, specifically, the cerebellum has a pivotal role in continuously adapting visual gaze [3]. A recent study [4] also reported that VOR abnormalities can be associated with central vestibular lesions in the vestibular nucleus, nucleus prepositus hypoglossi, flocculus, or with diffuse cerebellar lesions. All these deficits could lead to a specific abnormal HIT pattern; consequently, better examination of such HIT abnormalities may help clinicians to improve the knowledge on the effect of central and peripheral vistibulopathies.

For instance, Kheradmand & Zee [5], combining lesion and physiologic studies, described the cerebellar regions closely related to ocular motor functions (the VOR, the pursuit, and saccadic eye movements). Neurons in the vestibulo-cerebellum (i.e., a region of the cerebellum found in the flocculonodular lobe that receives vestibular and visual information) are activated in relation to head or target motion, during eye fixation, and in vestibular responses to head motion. More precisely, neurons located in the flocculus/paraflocculus are responsible for high-frequency (brief) vestibular responses, while the nodulus and the ventral uvula modulate the velocity-storage mechanism for low-frequency (sustained) vestibular responses, allowing a correct VOR response and reducing the nystagmus that normally occurs.

For several years, the cerebellum has been considered a key brain actor in neurodevelopmental disorders (NDDs) [6,7]. Some of the symptoms, such as social communication deficits, executive dysfunctions, poor motor control, or memory impairments, reported in autism spectrum disorders (ASD), dyslexia, and attention deficit/hyperactive disorders (ADHDs) may be related to functional deficits in distinct cerebellar sub-regions [8,9,10,11]. Some studies reported the presence of vermal morphologic abnormalities in subjects with ASD, a right cerebellar hypoperfusion in subjects with dyslexia, and a volume reduction of the posterior vermis or of the whole cerebellum in subjects with ADHD [12,13,14].

In the present study, our goal was to further explore the VOR in the specific context of children with NDDs. The fHIT was developed to estimate the VOR during passive head impulses. In other words, the VOR is tested by asking the subject to identify an optotype briefly presented during passive head impulses [15,16,17]. Compared with HIT, the fHIT offers a functional (recognition of the optotype) rather than quantitative result; in fact, in patients with vestibular symptoms, it is frequent that the gain of HIT returns to the normal range, while the fHIT continues to highlight a recognition deficit of the optotype [16,17].

Based on previous findings [16,17], we hypothesized that functional VOR (F-VOR), more than the quantitative VOR (Q-VOR), could be affected in children with NDDs. Besides the qualitative impact on the VOR in children with NDDs, we also hypothesized that the VOR parameters could be more severely affected in children with ASD than those with ADHD and finally those with reading impairments. Remember that the diagnosis of children with NDDs is quite difficult and is based on subjective tests only. Our goal is to develop new technique and objective tool to improve the NDD diagnosis; indeed, at least for children with ASD and ADHD, the diagnosis is based on subjective assessment only and the fHIT could be used as biomarker in the diagnosis of these pathologies.

## 2. Materials and Methods

### 2.1. Subjects

Four different groups of sex-, intelligence quotient (IQ)-, and age-matched children participated in the study (Table 1): *Group 1* included twenty children with reading impairments; *Group 2* enrolled twenty children with ADHD; *Group 3* included twenty children with autism spectrum disorder (ASD), but without intellectual deficiency (ID); while *Group 4* had twenty children with typical neurodevelopment (TD). Subjects from *Groups 1*, *2*, and *3* were enrolled in the study at the Child and Adolescent Psychiatry Department, Robert Debré Hospital (Paris, France). To be included, they should have a neurological exam in the normal range and should be naïve of psychotropic treatment. Children with reading impairment were recruited from the Centre for Language and Learning Disorders, to which they had been referred for a complete evaluation of their difficulties, including an extensive examination of their phonological capabilities. For each child, the time required to read a text passage, text comprehension, and the ability to read words and pseudo-words using the L2MA battery (oral language, written language, memory, attention [18]) were measured. The diagnosis of ADHD was done according to DSM-5 (Diagnostic and Statistical Manual of Mental Disorders) criteria [19] and carried out using the Kiddie-SADS semi-structured interview (Kiddie Schedule for Affective Disorders and Schizophrenia [20]). ADHD symptom severity was assessed using the ADHD rating scale parental report (ADHD-RS). This scale is based on a large collection of normative data and has demonstrated reliability and discriminant validity in children and adolescents [21,22]. Children with ASD were evaluated by the Expert Centre for ASD without ID; the diagnosis of ASD was based upon evaluation data from the ADI-R (Autism Diagnostic Interview-Revised [23], the ADOS (Autism Diagnostic Observation Schedule [24]), and expert clinical judgment based on DSM-5 criteria. To avoid a confounding effect of comorbidity on the main diagnosis, subjects with a comorbid diagnosis of ASD or ADHD were excluded from group 1, subjects with a comorbid diagnosis of ASD or reading impairment were excluded from group 2, and subjects with a comorbid diagnosis of ADHD or reading impairment were excluded from group 3. The mean intelligence quotient (IQ) was evaluated using the Wechsler Intelligence Scale for Children, fifth edition [25], for all subjects enrolled in groups 1 to 3. For all them, the IQ total score was in the normal range, i.e., between 85 and 115. The IQ of children with typical development was estimated using two subtests of the Weschler scale, one assessing his/her verbal ability (the similarities test) and one assessing his/her performance ability (matrix reasoning test). Exclusion criteria were as follows: *(i)* the presence of any binocular visual deficit, such as strabismus or high phoria (>6 PD exophoria, or any esophoria); and *(ii)* the presence of any vestibular impairment as vertigo, dizziness, or evident balance deficit.

The clinical characteristics of all four groups of children are summarized in Table 1.

The investigation followed the principles of the Declaration of Helsinki; the study was approved by the Institutional Human Experimentation Committee in France at the Hotel-Dieu hospital (INSERM-CEEI-IRB, n°16-290). Written informed consent was obtained from children and their parents after the nature of the procedure was explained.

### 2.2. Functional Head Impulse Test (fHIT)

**Figure 1:**
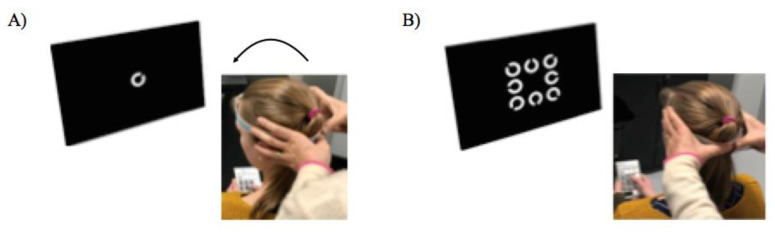
Experimental set up of the functional head impulse test (fHIT). (**A**): The child is positioned in front of a PC’s black screen (at 1.5 m) with a gyroscope fixed on the head by a head band. The child is invited to fixate on a small cross (not shown) presented on the center of the PC’s screen. A trained operator make head impulses in the lateral plane randomly to the right or to the left and a white Landolt C optotype appears eighty ms after head velocity surpasses 10 deg/s. (**B**): Then, eight different orientations of the Landolt C optotype are shown on the PC’s screen, and the child is instructed to select the correct position on a keypad.

This test, commercialized by BeOnSolutions society (www.beonsolutions.it), is based on the ability of a subject to read an optotype briefly presented during impulsive head rotations at varying angular accelerations [26]. Several studies have been conducted already using this test in adults from the general population [15,27] or with vestibular neuritis [16,28]. Briefly, the testing procedure was as follows: the child was seated on a chair placed at 150 cm distance from a computer screen connected to the fHIT device (Figure 1).

First, the static visual acuity was assessed using distance-scaled white Landolt C optotypes displayed on screen. All children had a normal visual acuity of 0.2 logMAR. As a consequence, this size was used in the experiment. During the test, childen had a head mounted gyroscope to measure head angular velocities in order to be sure that the head velocity reached optimal values to test the VOR (more than 150 deg/s). A trained operator performed subsequent head impulses, consisting of brief, small rotatory movements executed with both hands on the head of the child in the plane of each semi-circular canals pair and, at the same time, the child was asked to recognize the Landolt C optotype that was presented on a black PC screen for 80 ms in eight possible orientations (Figure 1A). Note that this time was based on preliminary tests done on children; moreover, several studies have already been done with adults using the same experimental set up used in the present study. Furthermore, it has been shown that subjects with normal vestibular function are able to stabilize the image and perfectly recognize the optotype with 80 msec target duration. The child had to recognize the Landolt C orientation and report it on a keypad showing all possible ring eight orientations (Figure 1B). The operator performed a minimum of 10 head impulses in each direction for each semi-circular canal plane (the left and right horizontal, left anterior and right posterior, and right anterior and left posterior directions). Normally, the subject is able to respond correctly, that is, to obtain 100% of correct responses. Before running the experiment, some trials were done for each direction of each semi-circular canal in order to be sure that child understood the test correctly. The software allows to evaluate, through visual feedback, the correct orientation of the vertical canals during the test execution and the correct acceleration imposed on the patient’s head. Between the tests of each semi-circular canal, a break of a few minutes was done in order to avoid fatigue. Note that, to avoid fatigue, the child had few breaks during the 10 trials; for this reason, we did not find any difference between the first 5 trials and the other one.

### 2.3. Data Analysis

Data were divided into 1000 deg/s^2^ wide bins based on the peak head angular acceleration reached during each head impulse that triggered the display of the Landolt C. Thresholds ranged from 2000 to 7000 deg/s^2^ and the data were thus organized in six bins for each subject. The fHIT software automatically separated the trials (i.e., the head impulses) according to the acceleration bins defined above and the semi-circular canal stimulated. As established by the fHIT procedure, the performance of a subject was assessed estimating the proportion of appropriated answers, that is, when the child recognized the correct orientation of the Landolt C [15].

### 2.4. Statistical Analysis

Statistical analysis was performed with the Statistica software (IBM SPSS Statistics 19, Armonk, NY, USA) using the GLM (Advanced Linear Models) with the four groups of children as inter-subject factors, and the percentage of the correct responses for the distinct semi-circular canals stimulated in the two directions (left and right) as within-subject factors. In the case of significant effects, we conducted post-hoc comparisons using Bonferroni correction for multiple comparisons. The effect of a factor was considered significant when the corrected *p*-value was below 0.05.

## 3. Results

**Figure 2:**
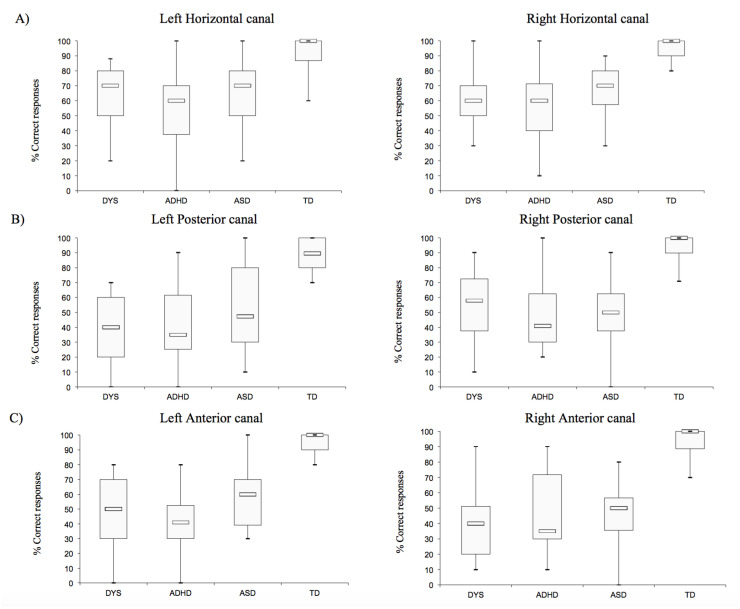
Boxplot with median and inter-quartile range of corrected answer of the Landolt C optotypes in the left- and rightward direction for each vestibular canal (**A**): horizontal, (**B**): posterior, and (**C**): anterior) for each child tested. DYS, children with dyslexia; ADHD, children with attention deficit hyperactivity disorder; ASD, children with autism spectrum disorders; TD, children with typical development.

We first explored the percentage of correct answers in the left- and rightward direction for each vestibular canal (horizontal, posterior, and anterior, respectively) for each group of children (Figure 2). We observed a significant group effect (*F*_(3,76)_ = 67.48, *p* < 0.0001). Bonferroni post hoc test showed that the performance of the fHIT of children with TD was significantly better than those of the three groups of children with NDDs (all *p* < 0.0001).

We also estimated the effect of the type of vestibular canal tested (*F*_(2,152)_ = 24.11, *p* < 0.0001) on the performance. Bonferroni post hoc analysis revealed that the percentage of correct responses of the horizontal vestibular canal was higher than the percentage of responses reported after posterior and anterior stimulations of the vestibular canals (both *p* < 0.01).

Finally, the analysis of variance (ANOVA) exhibited two significant interactions (vestibular canal × direction, *F*_(2,152)_= 10.89, *p* < 0.0001 and vestibular canal × group, *F*_(6,152)_ = 2.21, *p* < 0.04, respectively).

The Bonferroni post hoc analysis showed that the number of appropriated responses for both directions (left and right) of the horizontal vestibular canal was significantly higher with respect to the two directions of the posterior and anterior canals (all *p* < 0.01).

The performances measured for the posterior and anterior canals in the children with NDDs were significantly lower than those recorded in the horizontal canal (both, *p* < 0.001).

## 4. Discussion

The aim of the present study was to explore the VOR using the fHIT in children with NDDs in order to develop a new technique and objective tool to improve NDD diagnosis. We reported the following: *(i)* that children with neurodevelopmental disorders (NDDs) showed lower correct responses during the fHIT when compared with children with neurotypical development (we failed to report any significant difference between the three subgroups of children with NDDs); *(ii)* that all children reported higher correct responses for the horizontal semi-circular canal with respect to the other two semi-circular canals (posterior and anterior).

We compared the performance on this test to estimate a potential functional defect of the loop involved in the VOR, i.e., the vestibular and ocular systems, and the cerebellum [5]. As hypothesized, we observed that children with reading impairments, ADHD, and ASD had significantly poor performance on the fHIT. However, we were unable to identify a quantitate graduation between the potential severity of the phenotype and the impact on the VOR.

This finding is in line with previous studies suggesting deficiencies in vestibular capabilities in cognitive dysfunction and psychiatric disorders [29] and in children with NDDs. The corner piece of the vestibular system in NDDs has been stressed in a recent meta-analysis [30], which reported vestibular dysfunctions in children with intellectual disability (ID), ASD, ADHD, and specific learning disorders. These authors reviewed twenty studies in which central and/or peripheral vestibular deficiencies have been reported. For instance, Carson et al. [31], measuring the rotational vestibulo-ocular reflex (rVOR) in children with ASD, reported increased rVOR gain, which is the ratio of eye velocity to head velocity, indicating a possible lack of cerebellar inhibitory input to brainstem vestibular nuclei in this population and suggesting the presence of central vestibular deficiency in children with ASD. Note also that Deroualle and Lopez [32] reported several behavioral and neuroimaging studies suggesting the vestibular contribution to emotional and social cognition. Furthermore, Isaac et al. [33] reported reduced cVEMPs (cervical vestibular evoked myogenic potentials) amplitudes or a total lack of responses in children with ADHD, suggesting the presence of peripheral and central vestibular impairments in these children. Note that, even if cervical and ocular VEMPs have mostly been related to peripheral vestibular disorders, the characteristics and the diagnostic values of VEMPs have been used to assess the function of the central otolithic pathways; indeed, the cervical VEMPs are mediated by vestibular nuclei and uncrossed medial vestibulospinal tract descending in the lower brainstem and spinal cord [34].

Interestingly, Van Hecke and colleagues [30] pointed out that the majority of the studies were mainly based on the assessment of the horizontal semi-circular canals alone, which did not fully report the whole aberrations of the vestibular system. Recall also that some studies [35,36] observed the presence of the asymmetrical tonic neck reflex (which is a primitive reflex found in newborn humans that normally vanishes around 6 months of age) in children with ADHD, with dyslexia, and with emotional and behavioral difficulties. All these authors encouraged the community to develop a complete vestibular test battery to further characterize the vestibular capability in children with NDDs. In this line, we decided to use the fHIT, which allowed an extensive exploration of the qualitative vestibular functions. We thus observed that the VOR abnormalities were not similar in the distinct directions, and unequally affected the semi-circular canals; the best response was observed in the horizontal semi-circular canals than in the other two semi-circular canals (posterior and anterior). This result could be explained by the fact that horizontal canal is more frequently used, also in accordance with the study of Hullar [37], suggesting that the horizontal canal is particularly used during extrapolation of information to maintain good postural control. Contrary to our hypothesis, we have not identified patterns of abnormalities to discriminate the three groups of patients. The VOR abnormalities we reported allowed us to differentiate the group of affected patients versus controls. This suggests that the VOR abnormalities were more related to phenotypic or mechanistic characteristics shared by the three types of NDDs. Numerous studies have exhibited the link between vestibular function and several domains of visuospatial ability, which included spatial memory, navigation, mental rotation, and mental representation of three-dimensional space [38]. Substantial reports also suggested the vestibular system had an impact on attention and executive functions [39,40]. At the mechanistic level, one can hypothesize that the poor capability of vestibular system to compensate the head movements in children with NDD we observed in our study could be linked to shared abnormal regulation of the VOR, specifically by the cerebellum in NDDs [41,42]. In fact, the peripheral vestibular system, including semicircular canals and otolithic organs, projects to the vestibular nuclei located in the dorsal medullary brainstem; in turn, the neurons in the vestibular nuclei provide sensory input to the cerebellum, oculomotor nuclei, and spinal cord [43]. The cerebellum is one of the most consistent brain structures associated with ASD, gathering evidence from genetic, animal model, post-mortem, and neuroimaging studies [7,44]. For example, grey matter reductions were consistently reported in right Crus I, left lobule VIII, and medial IX, which may have specific long-term impacts on specific brain pathways involved in social communication abilities or executive functions in ASD [45,46]. As in ASD, several studies pointing out the pivotal role of the cerebellum in ADHD smaller cerebellar volumes have been reported [12], with a strong correlation between ADHD symptoms and the degree of reduction [47]. Additional abnormalities of the cerebellum have been reported in ADHD such as grey matter reductions in lobule IX and functional cerebellar dysconnectivity [48]. Finally, in subjects with reading impairment, structural and functional neuroimaging studies were also reported in the cerebellum. Reduced grey matter reductions have been reported consistently involving the left and right lobules VI [49].

Finally, we have to point out that the fHIT testing the VOR abilities confirms our hypothesis of poor cerebellar abilities in children with NDDs, even if imaging studies combined with eye movement recordings and quantitative vestibular tests will need to be done in such child populations to confirm our hypothesis.

Taken together, all these findings underlined the importance of systematically introducing the assessment of vestibular abilities (even indirectly tested) in children with neurodevelopmental disorders together with other clinical examinations of sensory and motor capabilities [50,51] in order to improve the diagnosis of these patients.

Finally, we could suggest that vestibular/cerebellar rehabilitation could help children with NDDs to develop compensatory mechanisms in order to override/decrease their pathologies. This hypothesis will be tested in future studies.

## 5. Limitation

This study reported a “qualitative” VOR deficit in children with NDDs. Note, however, that some NDD children had a correct response similar to TD children. Consequently, further studies with video-HIT could be necessary to explore the “quantitative” VOR capabilities in a larger population of NDD children. In the future, we could test together dynamic visual acuity and gaze stabilization capabilities in children with NDD in order to better understand the relationship between visual and vestibular abilities in these children. Finally, it will be also interesting to explore, in a larger number of NDD children, the eventual correlations between the fHIT response and clinical evaluation in order to more precisely define biomarkers in NDDs.

## 6. Conclusions

VOR abnormalities estimated using the fHIT could be used as a proxy for NDD impairments in children. Additional efforts will be required to better understand the intimate links between vestibular abnormalities and brain developmental disorders. Despite considerable efforts that have been made to identify biomarkers in NDDs, the results continue to be contrasting. The machine-assisted approach of neuroimaging/electrophysiological data may provide new directions to NDD research and leads to the identification of patterns of clinical, cognitive, and biological features, helpful for diagnosis and treatment. Qualitative-VOR abnormalities, which are easily measurable in patients with NDDs, could be integrated into these statistical models. In the future, we could explore the eventual benefits of vestibular rehabilitation in these kinds of patients.

## Figures and Tables

**Table 1:** Clinical characteristics (mean, standard deviation of the test score) in four groups of children tested (DYS, children with dyslexia; ADHD, children with attention deficit hyperactivity disorder; ASD, children with autism spectrum disorders; TD, children with typical development). For the L2MA test done in children with dyslexia, the standard deviation from normal mean is reported. ADHD-RS, ADHD rating scale parental report; L2MA, oral language, written language, memory, attention.

	*Group 1* DYS *N = 20*	*Group 2* ADHD *N = 20*	*Group 3* ASD *N = 20*	*Group 4* TD *N = 20*
**Age (years)**	9.6 ± 0.2	9.5 ± 0.3	9.7 ± 0.5	9.2 ± 0.4
**ADHD-RS total score**	5.1 ± 1.8	38.7 ± 1.7	5.2 ± 1.1	4.8 ± 1.2
**L2MA standard deviation from the mean**				
*Oral Language*	2.8			
*Written Language*	2.6			
*Memory*	2.7			
**Autism Diagnostic Interview-Revised (ADI-R) scores**				
*Social Reciprocal Interaction*			18.5 ± 1.5	
*Communication*			12.5 ± 0.9	
*Stereotyped Patterns of Behaviors*			5.1 ± 0.5	
**Autism Diagnostic Observation Schedule (ADOS) scores**				
*Social Reciprocal Interaction*			8.4 ± 0.8	
*Communication*			3.9 ± 0.4	
**Wechsler scale (WISC-V) scores**				
*Verbal Comprehension subscale*	101 ± 6	100 ± 5	101 ± 2	
*Perceptual Reasoning subscale*	99 ± 4	98 ± 3	97 ± 2	
*Working Memory subscale*	93 ± 3	91 ± 4	86 ± 4	
*Processing Speed subscale*	89 ± 3	90 ± 5	91 ± 3	
*Similarity test*	12 ± 1	12 ± 2	10 ± 2	12 ± 1
*Matrix reasoning test*	11 ± 1	10.2 ± 1	10.5 ± 1	10.8 ± 2
